# From neck pain to neurological deficit: Cervical tuberculous spondylodiscitis unveiled: A case report

**DOI:** 10.1016/j.ijscr.2025.112136

**Published:** 2025-11-01

**Authors:** Faten Limaiem, Mouadh Nefiss, Ramzi Bouzidi

**Affiliations:** a University of Tunis El Manar, Faculty of Medicine of Tunis, Tunisia; b Pathology Department, Hospital Mongi Slim La Marsa, Tunisia; c Department of Orthopedic Surgery, Hospital Mongi Slim La Marsa, Tunisia

**Keywords:** Spine, Cervical, Tuberculosis, Spondylodiscitis, Pathology, Case report

## Abstract

**Introduction:**

Cervical tuberculous spondylodiscitis is an uncommon but potentially devastating form of spinal tuberculosis. Its rarity, subtle clinical onset, and proximity to critical neurovascular structures make early diagnosis particularly challenging. Delayed recognition can lead to catastrophic outcomes, including spinal instability and neurological compromise. This case report aims to underscore the diagnostic pitfalls, highlight key imaging findings, and outline an effective management approach.

**Case presentation:**

A 36-year-old woman presented with a one-year history of progressive neck pain, followed by paresthesias in both upper limbs and the left lower limb. Examination revealed a suboccipital mass and sternocleidomastoid contracture. Imaging showed advanced destruction of the C1–C2 joint, with 12 mm diastasis, 80 % narrowing at the craniovertebral junction, and spinal cord compression. The patient underwent emergency posterior decompression and occipitocervical fusion. Histopathology confirmed tuberculosis through caseating granulomas and Langhans giant cells.

**Clinical discussion:**

The insidious onset and nonspecific symptoms of cervical spinal tuberculosis often delay diagnosis. MRI is essential for early detection of marrow edema, abscesses, and cord compression, while CT aids in assessing bone destruction. In this case, surgical intervention was required due to atlantoaxial instability and neurologic compromise. A multidisciplinary approach combining surgery and prolonged anti-tubercular therapy led to favorable outcomes.

**Conclusion:**

This case underscores the importance of early imaging, clinical vigilance, and timely surgical stabilization in preventing irreversible neurological damage. It contributes to the limited literature on upper cervical tuberculosis and highlights the value of individualized management strategies.

## Highlights

• Cervical tuberculous spondylodiscitis is rare but carries a high risk of neurological complications.
• Nonspecific early symptoms often delay diagnosis until advanced disease.
• MRI is crucial for early detection; CT assists in surgical planning.
• Histopathology confirms the diagnosis, supported by microbiological and molecular tests.
• Management combines anti-tubercular therapy with timely surgery.

## 1 Introduction

Spinal tuberculosis (TB), or Pott's disease, constitutes the most frequent musculoskeletal manifestation of TB, representing approximately 1 % of global tuberculosis cases. Among the various spinal regions, the thoracic and lumbar spine are most frequently involved, while cervical spinal TB is exceedingly rare, comprising only 3–5 % of cases [[1], [2], [3]]. This rarity is compounded by the unique anatomical and biomechanical characteristics of the cervical spine, including its narrow spinal canal, proximity to critical neurovascular structures, and high mobility, which render this region particularly vulnerable to severe complications such as spinal instability, deformity, and neurological deficits [4,5]. Advanced imaging, particularly MRI, plays a pivotal role in overcoming the diagnostic challenges posed by the insidious and nonspecific symptoms of cervical spinal TB. MRI's high sensitivity and specificity enable early detection of vertebral destruction, abscess formation, and neural compromise, facilitating timely medical or surgical intervention to prevent catastrophic outcomes. Despite its clinical significance, cervical spinal TB remains understudied, with limited literature specifically addressing its diagnostic and therapeutic challenges. Furthermore, the lack of standardized management protocols for this rare condition creates uncertainty in clinical decision-making, particularly regarding the timing and extent of surgical intervention. This report highlights a rare case of atlantoaxial TB spondylodiscitis with severe cord compression, emphasizing diagnostic challenges and the role of timely intervention.

This case report adheres to the SCARE Criteria [6].

## 2 Case presentation (Table 1)

### 2.1 Patient history and presenting complaint

A 36-year-old woman with an unremarkable medical history presented with a one-year history of progressively worsening neck pain. Over time, she developed paresthesias in both upper limbs and the left lower limb. She denied any history of trauma or systemic illness. Additionally, there was no known exposure to tuberculosis.

**Table 1:** Timeline of clinical events.

Time point	Event	Key findings/interventions
12 months before admission	Onset of neck pain	Progressive neck pain, worsening over time
6 months before admission	Development of neurological symptoms	Paresthesias in both upper limbs and left lower limb
Day of admission	Initial Evaluation	Temperature: 37.2 °C; Positive Hoffmann's sign on the left side; contracture of sternocleidomastoid
Day 1	Laboratory Workup	Hb: 9.9 g/dL, WBC: 13,400/mm^3^, CRP: 56 mg/L, blood glucose: 3.5 g/L
Day 1	Imaging Studies	Cervical spine CT: C1–C2 joint destruction, prevertebral and retropharyngeal fluid-filled cavities
Day 1	MRI Evaluation	C1–C2 dislocation with 12 mm diastasis, 6 mm spinal canal narrowing, bulbomedullary compression
Day 1	Chest CT	No pulmonary TB findings
Day 3	Surgical Intervention	Posterior surgical stabilization, C1 arch resected, occipito-cervical fusion with C3–C4 screws
	Intraoperative Biopsy	Biopsy samples collected for pathology
1-week post-op	Pathology Results	Histopathology confirmed TB: caseating granulomas, Langhans giant cells
Post-surgery	Initiation of Anti-Tubercular Therapy	Anti-TB therapy initiated, continued per protocol
Ongoing	Follow-up	Patient monitored for treatment response and neurological status

### 2.2 Physical examination findings

At the time of admission, the patient's body temperature was 37.2 °C. Neurological examination revealed intact sensation to light touch and pinprick, and symmetrical muscle strength. However, the patient reported persistent paresthesias in both upper limbs and the left lower limb. A positive Hoffmann's sign was observed on the left side, while deep tendon reflexes were symmetrical and preserved. The orthopedic examination showed contracture of the sternocleidomastoid muscle, along with a firm, non-tender suboccipital mass fixed to the deeper tissues. No signs of local inflammation, cervical lymphadenopathy, or tenderness along the thoracolumbar spinous processes were noted.

### 2.3 Diagnostic workup

**Fig. 1:**
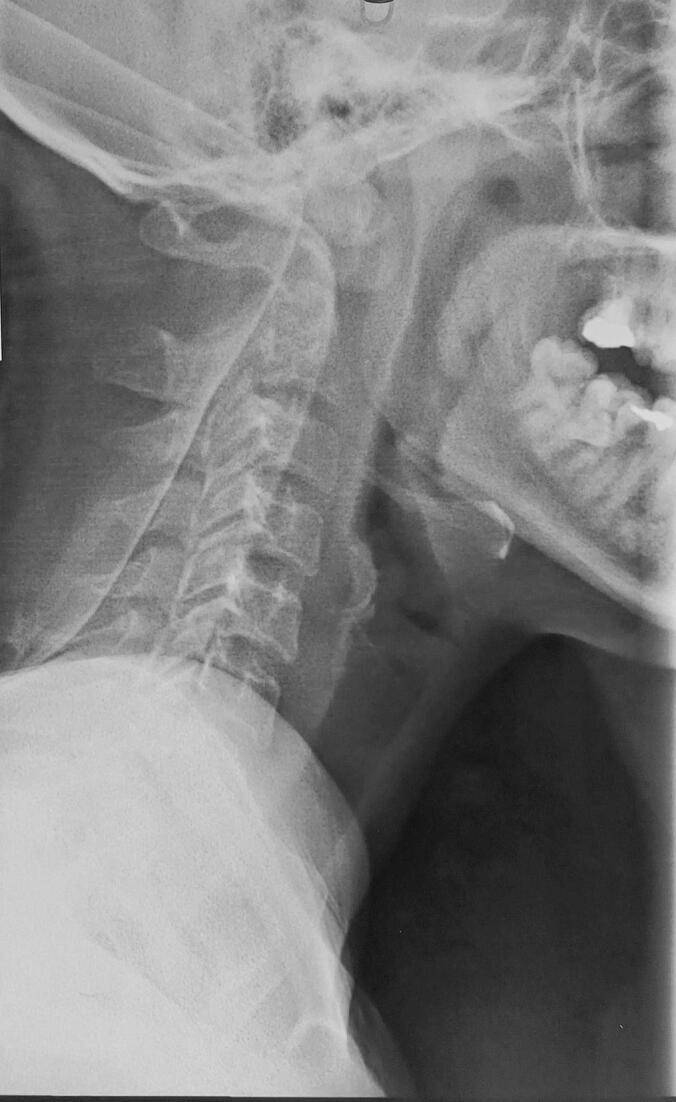
Lateral radiograph of the cervical spine showing marked atlantoaxial dislocation with anterior displacement of the atlas (C1) relative to the axis (C2). There is abnormal widening of the atlantodental interval, suggesting atlantoaxial instability. The alignment of the subaxial cervical vertebrae appears preserved.

**Fig. 2:**
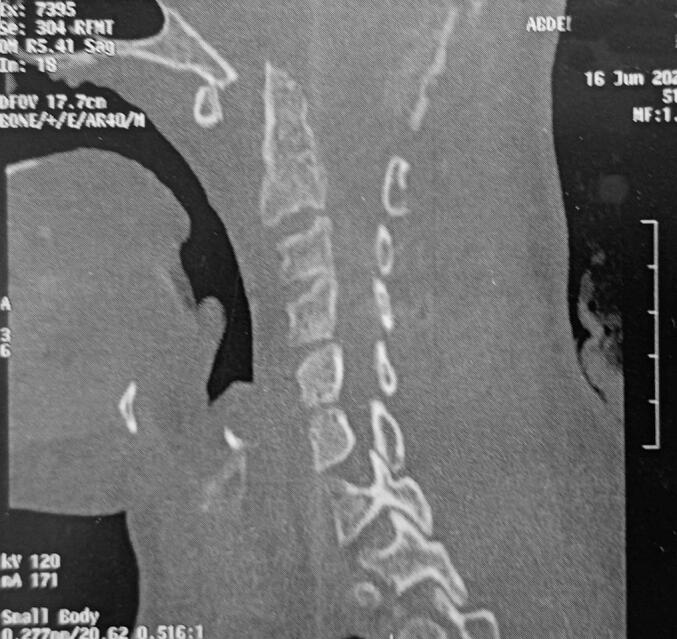
Sagittal CT reconstruction of the cervical spine showing atlantoaxial dislocation with anterior displacement of the atlas (C1) relative to the axis (C2). There is associated bony destruction and irregularity of the odontoid process.

**Fig. 3:**
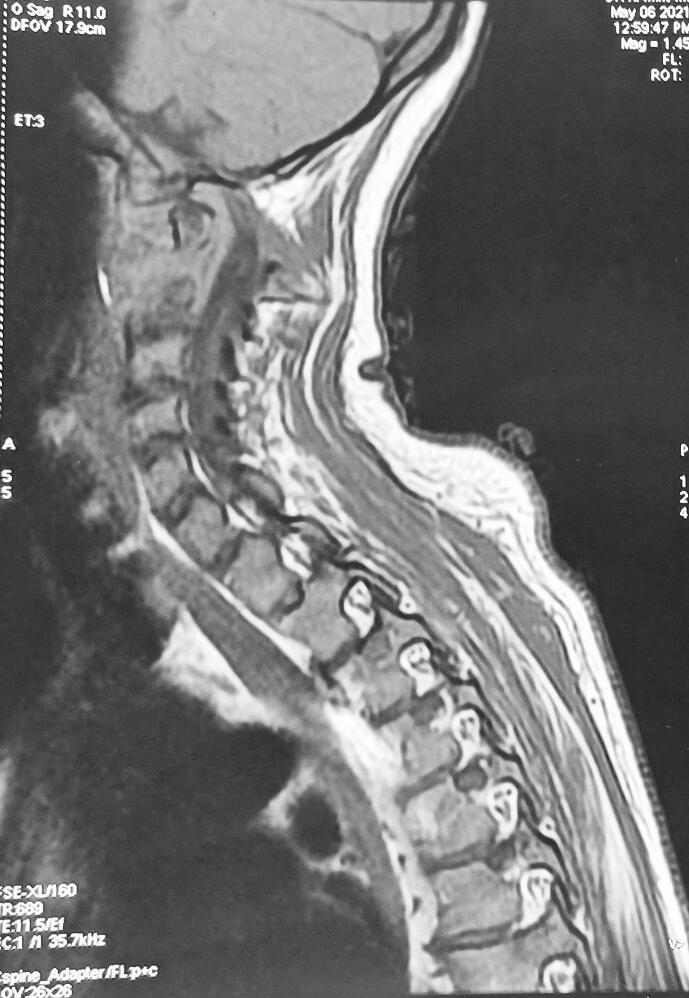
Sagittal T2-weighted MRI of the cervical spine showing marked atlantoaxial instability with anterior displacement of the atlas (C1) over the axis (C2). There is significant narrowing of the spinal canal at the craniovertebral junction, resulting in spinal cord compression. The spinal cord appears deformed at this level, and a hyperintense signal change within the cord suggests edema or early myelomalacia. Degenerative changes and normal alignment are noted in the lower cervical spine.

Laboratory tests revealed anemia (hemoglobin 9.9 g/dL), leukocytosis (WBC count 13,400/mm^3^), and elevated CRP (56 mg/L). Liver and renal function tests were within normal limits. Blood glucose was elevated at 3.5 g/L. A plain radiograph of the cervical spine revealed significant atlantoaxial dislocation, characterized by anterior displacement of the atlas (C1) relative to the axis (C2) (Fig. 1). Additionally, there was an abnormal widening of the atlantodental interval, indicative of atlantoaxial instability (Fig. 1). Contrast-enhanced MRI of the cervical spine showed advanced C1–C2 joint destruction with multiple prevertebral and retropharyngeal fluid collections, the largest measuring 30 mm. There was intra-articular effusion at C1–C2, osteolysis of the odontoid head, and posterior atlantoaxial dislocation, resulting in approximately 80 % narrowing of the cranio-cervical junction and spinal cord compression (Fig. 2). MRI confirmed C1–C2 dislocation with anterior displacement of the C1 ring and 12 mm diastasis. The spinal canal was narrowed to 6 mm, compressing the bulbomedullary junction (Fig. 3). Signal abnormalities were noted in C1, C2, and the left half of C3 vertebral body. Chest CT scan showed no evidence of pulmonary TB.

### 2.4 Treatment and follow-up

The patient underwent posterior surgical stabilization. After exposure from the occiput to C4, the posterior arch of C1 was resected. Two occipital holes were prepared near the foramen magnum. A tricortical iliac crest graft and cancellous bone were harvested.

**Fig. 4:**
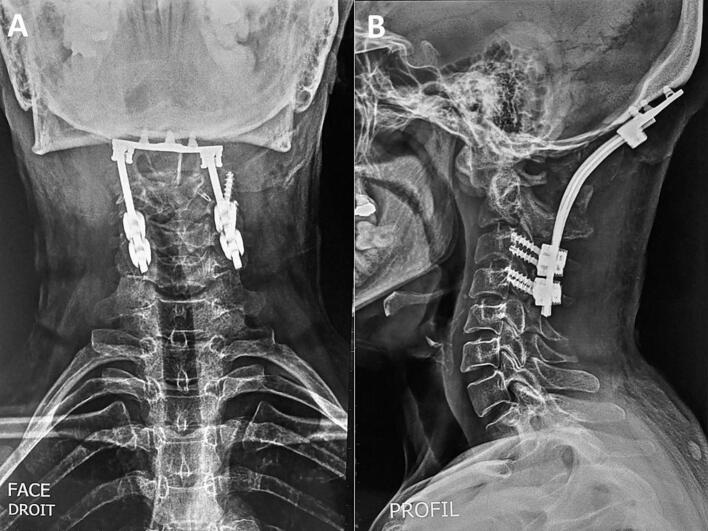
Postoperative cervical spine X-rays showing posterior occipitocervical instrumentation. (A) Anteroposterior view demonstrating bilateral occipitocervical fixation with screws and rods from the occiput to the upper cervical spine. (B) Lateral view illustrating the alignment of the occipitocervical junction and the position of the hardware spanning from the occiput to the cervical vertebrae (C2-C3).

An occipital plate was fixed with three 8 mm screws. Screws were placed bilaterally into the articular facets of C3 and C4 (Fig. 4). Following proper head positioning and alignment confirmation, fusion of C3–C4 was performed. The tricortical graft was placed near the foramen magnum, and a posterolateral bone graft using cancellous bone was also applied. Biopsy samples were collected during surgery.

### 2.5 Pathology findings

**Fig. 5:**
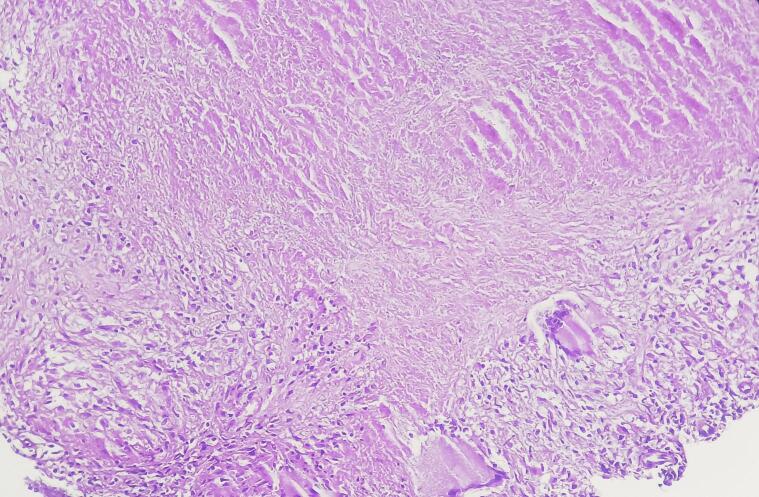
Histopathological section showing a granulomatous inflammatory reaction with caseous necrosis characteristic of tuberculosis. The central area of acellular, eosinophilic necrosis is surrounded by epithelioid histiocytes, Langhans-type multinucleated giant cells, and a peripheral rim of lymphocytes and fibroblasts. (Hematoxylin and eosin, magnification × 100).

Histopathological analysis confirmed the diagnosis of TB. Caseating granulomatous inflammation with epithelioid cells and Langhans-type giant cells was observed, consistent with tuberculous osteomyelitis (Fig. 5). Mycobacterial culture and PCR were not performed due to limited resources and lack of on-site molecular facilities at the time.

## 3 Discussion

Spinal TB remains the most common form of skeletal tuberculosis, accounting for nearly half of osteoarticular cases, followed by tuberculous arthritis and extraspinal osteomyelitis [[2], [3], [4], [5], [6], [7], [8], [9]]. It typically affects individuals between 40 and 50 years of age, with a slight male predominance [10]. While the thoracic and lumbar regions are more commonly involved, cervical spine involvement is rare, comprising only 3–5 % of spinal tuberculosis cases [3]. In a large case series, the cervical spine accounted for only 10 % of affected spinal levels, with the thoracic spine being involved in 45 %, the lumbosacral spine in 33 %, and multiple levels in 12 % [10]. In our case, the infection uniquely involved the atlantoaxial region (C1–C2), which represents a particularly rare form of cervical spinal tuberculosis, accounting for less than 1 % of all spinal tuberculosis cases [8,9]. The lateral masses of the atlas and the odontoid process of the axis are the most commonly affected structures, while involvement of the posterior elements, such as the neural arch or spinous process, is extremely rare [[10], [11], [12]]. In our patient, advanced joint destruction was observed at C1–C2, with radiological features showing a 12 mm diastasis and approximately 80 % craniovertebral junction stenosis, resulting in spinal cord compression hallmarks of a severe and progressive form of disease. The pathogenesis of cervical spinal TB typically involves hematogenous or lymphatic dissemination from a primary site, such as the lungs, gastrointestinal tract, or lymph nodes [2]. Rarely, the disease may result from direct extension of a chronic retropharyngeal abscess [2]. The radiographic absence of active pulmonary tuberculosis in our patient indicates that the cervical disease likely originated from either hematogenous seeding from a cryptic primary focus or the reactivation of a latent infection. This presentation of isolated spinal TB aligns with previous reports by Mahmoud et al. and Berker et al. [13,14]. Cervical tuberculosis typically manifests with an insidious onset of non-specific symptoms, primarily axial neck pain, stiffness, and restricted motion, which commonly contributes to a significant delay in diagnosis [13]. In our case, The patient's neck pain was distinguished from common etiologies by its progressive, one-year duration and the presence of “red flags,” including a fixed suboccipital mass, sternocleidomastoid contracture, and the evolution of myelopathic symptoms (paresthesias in three limbs). This constellation of findings pointed decisively away from benign mechanical pain and toward a destructive process, prompting advanced imaging that confirmed the diagnosis. This frequently results in clinical presentation only after the emergence of neurological compromise. Our patient's one-year history of progressive neck pain prior to developing paresthesias exemplifies this challenging clinical course. This pattern is consistent with the literature; Yin et al. reported a mean diagnostic delay of 4.5 months in their cohort [8], a finding further illustrated by Mahmoud et al. in a case involving a year-long history of neck pain preceding neurological deficits [13].

Tuli [15] and Whalen [16] also highlighted that neurological deficits are not uncommon in cervical spinal tuberculosis, especially at the C1–C2 level, with rates of cord compression and tetraplegia reaching 42 % and 15–20 %, respectively. Our patient's clinical presentation underscores a critical feature of early compressive myelopathy: the presence of significant subjective neurological symptoms in the absence of objective deficits on examination. The reported paresthesias represent *positive* sensory phenomena, likely caused by direct irritation and ectopic firing within the ascending sensory tracts of the compressed cord. These irritative symptoms often precede the development of *negative* phenomena, such as sensory loss or weakness, which indicate structural tract degeneration. In this context, the objectively observed Hoffmann's sign served as a crucial clinical bridge, providing definitive evidence of upper motor neuron pathology that corroborated both the patient's subjective complaints and the severe radiographic compression [1,15]. The clinical examination in our case revealed a firm, non-tender suboccipital mass and contracture of the sternocleidomastoid muscle findings that are rarely described in the literature and may correspond to cold abscess formation or subligamentous spread. Cold abscesses are seen in up to 70 % of spinal tuberculosis cases and may present in atypical locations depending on anatomical spread [7]. These lesions are characteristically painless and may lack signs of inflammation, further complicating early detection. Advanced imaging is crucial for the diagnosis and management of cervical tuberculous spondylodiscitis. While plain radiographs may appear normal in early disease and CT is superior for evaluating osseous destruction and subtle cortical erosions, MRI remains the gold standard, with sensitivity and specificity exceeding 90 % for detecting spinal cord compression, marrow edema, and abscess formation [[2], [3], [4], [5],^15^,^17^]. In our case, MRI was pivotal, revealing extensive C1-C2 joint destruction, a narrowed spinal canal measuring 6 mm, anterior displacement of the C1 ring, and signal abnormalities consistent with active inflammation and cord compression. In diagnostically challenging scenarios, FDG-PET imaging offers high diagnostic accuracy, enabling precise lesion localization and differentiation from other pathologies. This emphasis on advanced imaging is critical, as it facilitates timely intervention, thereby reducing morbidity and preventing severe neurological deficits. The imaging profile in our case shares commonalities with other reports; the severe cord compression mirrors findings by Jain et al. [1] at C6-C7 and Ekasari et al. [3] at C2-C4. However, the specific atlantoaxial involvement in our patient highlights the unique challenges of this region, where instability, rather than a solitary compressive mass, often dictates a distinct surgical approach. Laboratory findings, while non-specific, typically include elevated inflammatory markers. In our patient, leukocytosis and an elevated CRP of 56 mg/L were observed. The erythrocyte sedimentation rate is also frequently increased and is a useful parameter for monitoring therapeutic response [8]; its persistent elevation beyond three months may suggest treatment failure, poor compliance, or drug resistance. As first-line anti-tubercular therapy carries hepatotoxic risk, routine liver function monitoring is essential [1]. A definitive diagnosis, however, hinges on histopathological confirmation. In this case, the intraoperative biopsy was diagnostic, revealing caseating granulomas with Langhans giant cells. Although molecular tests like Xpert MTB/RIF and mycobacterial culture offer rapid, sensitive detection, histology remains critical, particularly for atypical presentations [[1], [2], [3]].

Differential diagnoses of destructive cervical spine lesions include brucellar spondylodiscitis, pyogenic infections, fungal osteomyelitis, and malignancies such as lymphoma, multiple myeloma or metastasis [5,6,17]. Compared to brucellar infection, spinal tuberculosis is more likely to produce cold abscesses, necrosis, and instability, as was evident in our case [5,6].

Medical therapy remains the cornerstone of treatment. The World Health Organization recommends a two-month intensive phase of four-drug therapy (isoniazid, rifampicin, ethambutol, and pyrazinamide), followed by a 7–9-month continuation phase with two or three drugs [2,3,8]. In our case, anti-tubercular therapy was initiated postoperatively and closely monitored. Response to treatment was assessed using serial ESR/CRP levels and clinical evaluation. Surgical intervention is required in selected cases, particularly in the presence of neurological deficits, spinal instability (e.g., atlantoaxial dislocation, vertebral collapse, or kyphosis ≥30°), large abscesses, or failure of medical therapy [2,3,8]. In our case, the combination of progressive neurological symptoms, advanced atlantoaxial destruction, and irreducible dislocation necessitated urgent surgical intervention. The patient underwent posterior decompression and occipitocervical fusion, a technique well-supported in the literature for upper cervical involvement. Shetty et al. recommend posterior stabilization in irreducible or unstable lesions of the craniovertebral junction [2], while Yin et al. reported excellent outcomes with posterior or combined approaches in patients with upper cervical tuberculosis [9]. Our surgical approach allowed decompression of the spinal cord, correction of the deformity, and stabilization of the craniovertebral junction. Tricortical and cancellous iliac crest grafts were used for fusion, and the fixation construct extended from the occiput to C4. This technique not only provided mechanical stability but also allowed biopsy sampling. Postoperative recovery was uneventful, and the patient continued anti-tubercular therapy with good compliance.

## 4 Conclusion

In conclusion, by contextualizing our patient's presentation and management within the framework of existing literature, this case report reinforces several key principles in managing upper cervical TB. The diagnostic delay and insidious onset observed here are common, underscoring the need for high clinical suspicion. The successful outcome achieved through a tailored surgical approach posterior occipitocervical fusion for atlantoaxial instability validates the findings of larger studies that advocate for individualized surgical strategies based on the specific anatomical and pathological characteristics of the lesion [4,5]. This case thus contributes valuable insights to the limited literature on this rare and severe form of spinal TB, emphasizing that a multidisciplinary approach combining timely imaging, histopathological verification, long-term chemotherapy, and judicious surgical intervention is indispensable for preserving neurological function.

## Consent

Written informed consent was obtained from the patient for publication of this case report and accompanying images. A copy of the written consent is available for review by the Editor-in-Chief of this journal on request.

## Ethical approval

Ethical approval was waived by the Hospital's Ethics Committee, as institutional policy does not require review for single-patient case reports that are not considered research.

## Guarantor

Dr. Faten LIMAIEM.

## Provenance and peer review

Not commissioned, externally peer-reviewed.

## Funding

This research did not receive any specific grant from funding agencies in the public, commercial, or not-for-profit sectors.

## Author contribution

Dr. Faten LIMAIEM: Conceptualized the study, drafted the initial manuscript, revised and edited all sections, and approved the final version for submission.

Prof. Mouadh NEFISS & Prof. Ramzi BOUZIDI: Critically reviewed and edited the manuscript, contributed to data acquisition and interpretation, and provided final approval for submission.

## Declaration of competing interest

None declared.
